# One-Year Follow-Up of Patients Undergoing Transvenous Extraction of Pacemaker and Defibrillator Leads

**DOI:** 10.1371/journal.pone.0144915

**Published:** 2015-12-22

**Authors:** Maciej Kempa, Szymon Budrejko, Marta Piepiorka-Broniecka, Jan Rogowski, Dariusz Kozlowski, Grzegorz Raczak

**Affiliations:** 1 Department of Cardiology and Electrotherapy, Medical University of Gdansk, Gdansk, Poland; 2 Department of Cardiology, St. Vincent a Paulo Hospital, Gdynia, Poland; 3 Department of Cardiac Surgery, Medical University of Gdansk, Gdansk, Poland; Nagoya University, JAPAN

## Abstract

**Introduction:**

The number of pacemaker and ICD implantations has increased substantially in the recent years. Therefore, complications are also observed in a greater number. In many cases, transvenous extraction of the previously implanted device (pacemaker or ICD) is the only solution. One may find in the literature information about the efficacy and safety of that procedure, but data concerning the results of long-term follow up are still limited.

**Aim:**

The aim of the study was to assess the one-year mortality in the cohort of patients undergoing transvenous lead extraction procedures in our centre.

**Methods:**

Records of the patients undergoing transvenous lead removal in the Department of Cardiology and Electrotherapy of the Medical University of Gdańsk were analyzed. We collected detailed information about 192 patients that had undergone the procedure from January 2003 until June 2012. Data were collected from medical and surgical records. We analyzed concomitant diseases, indications, and possible complications. Long-term follow-up data were gathered in the follow-up ambulatory records and over-the-phone interviews with patients or families. In several cases, we consulted the database of the Polish National Health Fund.

**Results:**

During the early post-operative period 5 patients died, although none of those deaths was associated with the procedure itself. No other major complications were observed. During one-year follow-up other 5 patients died, which gave the overall one-year survival rate of 92.7%. Heart failure, renal failure and an infective indication showed significant association with increased mortality.

**Conclusion:**

Results of transvenous lead extraction, a relatively safe procedure, should be assessed over time extending beyond the sole perioperative period. Some complications may be delayed in their nature, and may be observed only during the long-term follow up.

## Introduction

Constant increase of the number of pacemaker (PM) and cardioverter-defibrillator (ICD) implantations, as well as the increasing number of complications of cardiac electrotherapy, result in the growing amount of transvenous lead extraction (TLE) procedures. Lead removal procedures are subdivided into lead explant (with simple traction, without any specialized tools apart from normal stylet and screw retraction tool, usually possible with leads that have very short dwelling time) and lead extraction (removal of leads that have been implanted for more than one year or leads requiring any tools for their removal). Tools and techniques used for lead removal comprise: simple traction, traction devices (snares, lead locking devices), as well as different types of specialized sheaths—mechanical, laser, electrosurgical, and mechanical with rotating tip. The general idea and goal is to lock the lead tip with a lead locking device for appropriate stiffness and transfer of the point of force application from the lead connector to the lead tip, and then to dissect all the adhesions preventing lead removal with any sheath available or applicable. Current guidelines for TLE describe precise indications for such procedures [1]. The list of indications is very detailed and may not be cited in extension in this text, but in summary, transvenous lead extraction may be needed in case of systemic or local infection of the implanted cardiac electronic device, lead failure, vascular stenosis with the upgrading indication, and many other clinical situations.

The TLE consensus statement also contains clear definitions of possible complications and procedural outcomes. Outcome definition includes three categories: complete procedural success (removal of all targeted material without any permanently disabling complications or death), clinical success (removal of all targeted material or retention of a small portion of the lead that does not impact the goal of the procedure) and failure (inability to achieve either complete procedural or clinical success, or occurrence of any permanently disabling complication or procedure related death) [1]. Possible complications are subdivided into major (death, life threatening events, any event that causes significant disability, or any event that requires significant surgical intervention) and minor (all the remaining complications). Major complications include: death, cardiac or vascular avulsion or tear requiring intervention or surgical repair, pulmonary embolism requiring surgical intervention, respiratory arrest or anesthesia related complication leading to prolongation of hospitalization, stroke, and pacing system related infection of a previously non-infected site. Minor complications list is longer, and includes above all: pericardial effusion not requiring pericardiocentesis or surgical intervention, hemothorax not requiring a chest tube, hematoma at the surgical site requiring reoperation for drainage, arm swelling or thrombosis of implant veins resulting in medical intervention, vascular repair near the implant site or venous entry site, hemodynamically significant air embolism, migrated lead fragment without sequelae, blood transfusion related to blood loss during surgery, pneumothorax requiring a chest tube and pulmonary embolism not requiring surgical intervention [1]. Despite the complexity of indications, techniques and possible outcomes, transvenous lead extraction has become a nearly standard procedure in selected patients and in experienced centers for TLE, with major complication rate below 1% achievable in experienced hands. Nonetheless, data regarding long-term follow-up of patients after TLE procedures are limited.

The aim of our analysis is to assess the results of one-year follow-up of patients that were subject to transvenous extraction of pacing and defibrillating leads.

## Methods

We have analyzed data from the Electrophysiology Laboratory regarding all the implantation, replacement and extraction procedures of PM/ICD systems during the period from January 2003 until June 2012. Further analysis was conducted for patients undergoing TLE procedures. Because of the increasing number of those procedures in the recent years, that are also performed as emergency procedures for early complications of device therapy, (such as cardiac perforation or early system infection), patients operated on within the first year from the initial implantation procedure were also included in our analysis. We reviewed clinical data, such as comorbidities, indications for the procedure, dwelling time of the leads and their type, and the outcomes of the extraction procedure, including possible complications. Those data were subsequently joined with information about survival of patients after the procedure that was acquired from available medical records and trans-telephonic query of patients and their families. In case of patient’s death, details were gathered–if possible–from the available medical documentation.

Indications for the procedure, procedural outcomes and complications were qualified according to the classifications published in the Heart Rhythm Society Expert Consensus for TLE in 2009 [1]. Outcome was defined as ‘complete procedural success’ in case of complete removal of the lead and every part of it, unless persistent complications or patient’s periprocedural death occurred. ‘Clinical success’ was defined as extraction of the lead with a minor part of it remaining, with no influence on the pre-defined purpose of the procedure and no increase in the risk of perforation, embolism or infection. ‘Failure’ was attributed to any procedure that failed to achieve either complete success or clinical success, as defined above. Complications were divided into two categories: major (death, damage to the cardiovascular system requiring invasive treatment, pulmonary embolism requiring invasive treatment, stroke, complications of general anesthesia that prolonged hospital stay) and minor (pericardial effusion or hemothorax not requiring intervention, pocket hematoma requiring intervention, venous thrombosis, pulmonary embolism, pneumothorax, blood loss requiring transfusion) [1]. All data were processed with Microsoft Excel spreadsheet version 2007 and Statistica software version 8.5. Descriptive statistics for continuous variables were expressed as mean, median, standard deviation, and ranges. Discrete variables were expressed as frequencies and percentages. In all analyses, statistical significance was assumed for differences with p value below 0.05. Survival analysis and survival plots were calculated using the Kaplan-Meier method. Survival time in different groups was compared with the log-rank and F-Cox tests. Prognostic factor analysis was conducted with the use of proportional hazard model (Cox model), and variables in the model included the presence of coronary artery disease, prior myocardial infarction, chronic heart failure, chronic kidney disease, lead dysfunction, infective indication for the procedure, atrial fibrillation and arterial hypertension.

The study was approved by the Independent Bioethical Committee for Scientific Research at the Medical University of Gdansk, Poland. The study design was based on retrospective analysis of patients files and records and telephonic verification of patients’ status (long-term with respect to the date of the extraction procedure). Therefore a written consent could not be acquired. Patients (or in few cases of patient’s late death–their families) were informed at the beginning of the telephonic call about the scientific nature of the conversation, the identity of the researcher, the strict confidence of their personal and medical data, and were subsequently asked for oral consent. In case of denial we planned to exclude such patient’s data from our analysis, but there was no such a case. All patients (or relatives) interviewed gave their oral consent to include their medical data in the study. Because of the rectrospective nature of the study, which was based on the analysis of medical records, the Independent Bioethical Committee for Scientific Research at the Medical University of Gdansk approved the protocol of the study as described above, including the patient’s consent procedure.

### Lead extraction procedure

TLE procedures were performed in the operating room of the Electrophysiology Laboratory. Initial preparations included: patient’s informed consent, standard blood tests, bacteriological cultures of the wound and/or blood (if indicated), chest x-ray for lead inspection, and transthoracic echocardiography (and also transesophageal, if needed) to search for thrombi on the leads that would constitute contraindications for transvenous lead extraction. For each procedure, at least two units of compatible blood were prepared and cross-matched. The start of the procedure and its duration were reported to the department of cardiac surgery, to provide surgical stand-by in case of any complications. High-risk procedures were conducted in the operating room of the department of cardiac surgery, in general anesthesia, with a patient prepped for immediate emergency sternotomy and cardiosurgical repair procedure.

As a routine, procedures were performed in local anesthesia or sedation with an option of general anesthesia, if needed. Routine approach for lead removal was direct traction of the lead through the vein of lead insertion (mostly subclavian vein), or extraction using a lead locking device, if needed (Liberator, Cook), and telescopic teflon or polyurethane sheaths to dissect adhesions of the lead to the cardiovascular bed. If necessary, femoral approach was used with a so-called femoral station. From 2011 on we had a chance to use a dissecting sheath system (Evolution, Cook).

## Results

As a whole, we analyzed data of 4857 patients operated on in the Electrophysiology Laboratory during the time period of the study. In that cohort, 192 patients (136 M, 56 F) were subject to transvenous lead extraction procedures. Altogether, 268 leads were extracted: 181 pacing leads and 87 defibrillating leads. In 123 patients one lead per patient was extracted, in 63 cases—2 leads, in 5 cases—3 leads and in one case—4 leads. The mean time from lead implantation to its extraction was 42.7 months. The most common indication for the procedure was infection, either in the form of PM/ICD pocket infection (65 patients) or infective endocarditis (27 patients). Lead fracture/dysfunction was the main indication in 75 patients, upgrading indication in 15 patients, right ventricle perforation in 4 patients, lead dislocation in 3 cases, venous thrombosis in 2, and indication for radiotherapy in one case (Table 1).

**Table 1:** Detailed characteristics of the analyzed cohort.

Indication (number of patients)	Pocket infection (65)	IE (27)	Lead dysfunction (75)	Upgrading indication (15)	Other (10)
Sex					
Female	20 (31%)	6 (22%)	24 (32%)	3 (20%)	3 (30%)
Male	45 (69%)	21 (73%)	51 (68%)	12 (80%)	7 (70%)
Age (years)	65.0	59.1	63.4	74.2	57.2
Device type					
Pacemaker	46 (71%)	16 (59%)	20 (27%)	13 (86%)	6 (60%)
ICD	16 (25%)	11 (41%)	54 (72%)	1 (7%)	4 (40%)
CRT	3 (4%)	0	1 (1%)–CRT-D	1 (7%)–CRT-P	0
Concomitant disease					
DM	19 (29%)	10 (37%)	15 (20%)	9 (60%)	3 (30%)
CAD	23 (35%)	9 (33%)	36 (48%)	8 (53%)	7 (70%)
Post-MI	17 (26%)	6 (22%)	23 (31%)	7 (47%)	3 (30%)
CHF	24 (37%)	16 (59%)	46 (61%)	12 (80%)	4 (40%)
HA	37 (57%)	13 (48%)	34 (45%)	8 (53%)	5 (50%)
AF	22 (34%)	10 (37%)	27 (36%)	6 (40%)	5 (50%)
CKD	6 (9%)	10 (37%)	11 (15%)	4 (27%)	1 (10%)
EF	44.2%	41%	38%	30%	44.2%

Abbreviations: ICD–implantable cardioverter-defibrillator; CRT–cardiac resynchronization therapy device; DM–diabetes mellitus; CAD–stable coronary artery disease; post-MI–history of prior myocardiial infarction; CHF–chronic heart failure; HA–arterial hypertension; AF–atrial fibrillation; CKD–chronic kidney disease; EF–ejection fraction.

### Results of TLE

In 2 cases the procedure was concluded as failure. In the first case, an excess of lead body entering the right subclavian vein formed a displaced loop with adhesions in the internal jugular vein. That setting made it impossible to insert a locking stylet and use telescopic sheaths. The patient was treated by means of cardiac surgery with an unfavourable outcome–he died because of sepsis. In the second case, we managed to remove the pacing lead incompletely, with a small portion of it remaining in the subclavian vein (the distal part of the lead approximately 1cm in length, torn off at the ring location). The indication for the procedure was in this case pacemaker pocket infection, and the abovementioned situation has to be classified as failure. Nonetheless, 2 years later the patient underwent an ipsilateral implantation of a dual-chamber ICD. During 2 years of follow-up no reoccurrence of infection was noted in that patient.

Despite successful TLE procedure, 5 patients died in the postoperative period, during hospitalization related to the procedure. Death occurred on average on the 8th day after the procedure (range: 1–21 days). In 3 cases the direct cause of death was septic shock (death during the first 24 hours in 2 patients, the third patient died on the third day), and in 2 cases it was heart failure (death on the 14th and 21st day). Patients that died of septic shock were operated on because of systemic staphylococcal infection resistant to antibiotic therapy. In the remaining cases, in 5 patients the outcome was classified as clinical success, and the rest was full procedural success.

Apart from the 5 fatal outcomes described above, no other major complications were observed in the periprocedural period. Minor complications included: pocket hematoma in the empty pocket after device removal in 5 patients, minor pericardial effusion not requiring any intervention in 3 patients and pneumothorax in 1 patient.

### Long-term follow-up

Patients operated on and discharged from our clinic joined the follow-up programme for at least one year. In 38 cases, the follow-up of the period after discharge could not be completed. Nonetheless, based on the national healthcare insurance database we determined that 15 patients out of that group were dead, but detailed information about the time and direct cause of death was missing. Therefore, for the final analysis we selected 149 patients (102 M, 47 F) in the mean age of 64.3 years. The mean time of follow-up was 32 months (range 4–128). During the first year of follow-up 5 patients died: during the 4th, 5th, 7th, and two during the 8th month post-procedure. The cause of death was accordingly: relapse of infective endocarditis (IE), sudden cardiac death, stroke, heart failure and gastrointestinal hemorrhage. Until the end of the first year of follow-up, 26 patients remained without any implantable device. In that group 20 patients had had a pacemaker before extraction (13 DDD, 4 VVI, 2 AAI, 1 VDD) and 6 patients—ICD (DDD- 2, VVI– 4). Patients after pacemaker removal had a good tolerance of possible bradycardia. The reason to abandon ICD reimplantation was: improvement of left ventricle contractility and lack of indication for an ICD in 2 patients, recurrent IE in 1 patient, need for repeated radiotherapy in 1 patient, and 2 patients refused to undergo ICD reimplantation.

**Fig 1:**
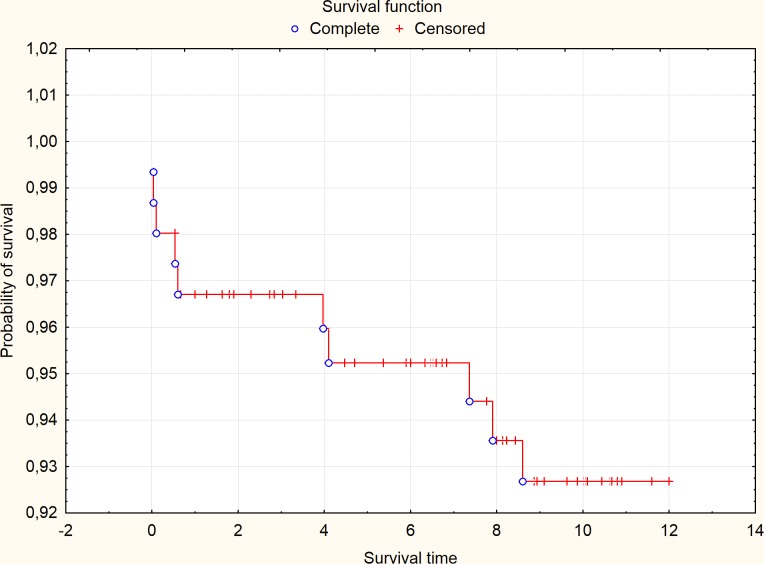
Probability of one-year survival in the analyzed cohort (time of survival in months).

Statistical analysis revealed the value of total annual survival of 92.7% (Fig 1).

Statistical analysis also showed that several factors had a significant influence on survival, and these were: indication for the procedure (infective vs non-infective) and chronic cardiac or renal failure. One-year survival was not influenced by the presence of coronary artery disease, prior myocardial infarction, arterial hypertension or atrial fibrillation (Table 2). The time range from lead implantation to its removal also did not influence survival.

**Table 2:** Analyzed factors suspected to influence one-year survival.

Factor	HR	P-value
CAD	0.304578	0.144589
Post-MI	0.241775	0.191491
CHF	3.868078	0.042159
CKD	3.995416	0.040590
Lead dysfunction	0.182038	0.108226
Infective indication for TLE	34.31708	0.042211
AF	2.715600	0.140498
HA	1.161774	0.836067

Abbreviations: CAD–stable coronary artery disease, post-MI–prior myocardial infarction, CHF–chronic heart failure, CKD–chronic kidney disease, AF–atrial fibrillation, HA–arterial hypertension.

## Discussion

Transvenous lead extraction procedure is often difficult to complete, although in experienced hands it occurs to be relatively safe. The frequency of serious intraoperative complications and death during the procedure is not very high, and in the majority of publications it remains below 4% and 2%, respectively, in most cases being even lower [2–7]. Nonetheless, one should note that in the postoperative period the risk of death is higher. Most publications that contain data of that type show the 30-days mortality rate of 2.1–2.7% [8–9]. Those results are concordant with our observations. In our cohort, 5 patients (2.6%) died during the first month post-procedure, and in none of those cases the cause of death was associated with intraoperative factors, nor was it the result of any medical error or complication of the procedure itself. In 3 cases death was a consequence of systemic staphylococcal infection, and in 2 cases–of severe heart failure. That finding reflects the fact, that Staphylococcus is one of the most frequent pathogens causing infective complications in patients with cardiac implantable electronic devices [10]. During the follow-up period of one year other 5 patients died, which results in the overall one-year mortality of 7.3%. That number is smaller than the result published by Maytin et al. of 8.4% [8]. Hamid et al. observed mortality of 6.6% during the follow-up lasting approximately 3 years [9]. The analysis of risk factors of death after TLE reveals high concordance of researchers’ opinions and data that a non-infective indication, and specifically systemic infection, is a key risk factor. In our cohort, among 10 patients with fatal outcome, 8 had that type of indication for the procedure (7—systemic infection and 1—pocket infection). Among 27 patients with systemic infection as many as 7 died, which constitutes 26%. That is half of the percentage published by Henrikson et al., but that group followed the patients for a longer period of time (the maximum of 55 months) [11]. Our result is in line with the observation of Maytin et al., who reported the annual mortality of 25% in a cohort of patients with systemic infection [8]. Other factors that increased one-year mortality in our cohort of patients undergoing TLE were: heart failure and renal failure. Those factors increased the risk of death almost fourfold. The risk of death was not increased by the history of coronary artery disease, prior myocardial infarction or arterial hypertension. In the publications cited above only renal failure was assessed from the group of factors that we included in our analysis. Unfavourable influence of that factor on mortality after TLE procedure is confirmed in those reports [8]. A somewhat surprising finding that coronary artery disease did not influence one-year survival in our cohort is consistent with other studies [8]. CAD is a risk factor of mortality in the general population, hence it might be speculated that group sizes of TLE patients are insufficient to reveal such a relatively subtle factor, compared to an infective indication, but this is highly uncertain, not recognized in the literature and requires further research.

It should also be kept in mind, that removing any device that was intended for death prevention (such as for example an ICD) might increase patient’s risk after the device removal. But that has not been the case in our cohort, because none of the secondary prevention patients was ever discharged from hospital (our clinic or the referring department) before they completed antimicrobial therapy and received appropriate protection again (which means usually a contralateral ICD in case of an infective indication for removal). Primary prevention patients were reevaluated for their risk and treated accordingly.

## Conclusion

Despite the safety of TLE procedure as assessed in short-term post-procedure, one should recognize that both complications and death of a patient may occur later in the course of the follow-up, especially in case of an infective indication. Therefore, qualification for the procedure should include long-term follow-up data of patients treated with that method.

## Supporting Information

S1 File Contains an MS Excel table with all relevant data gathered for our cohort. (XLSX)

## Abbreviations:

PM: pacemaker
ICD: implantable cardioverter-defibrillator
TLE: transvenous lead extraction
HRS: Heart Rhythm Society
